# Scaffold Library for Tissue Engineering: A Geometric Evaluation

**DOI:** 10.1155/2012/407805

**Published:** 2012-09-26

**Authors:** Nattapon Chantarapanich, Puttisak Puttawibul, Sedthawatt Sucharitpwatskul, Pongnarin Jeamwatthanachai, Samroeng Inglam, Kriskrai Sitthiseripratip

**Affiliations:** ^1^Institute of Biomedical Engineering, Faculty of Medicine, Prince of Songkla University, Songkhla 90110, Thailand; ^2^National Metal and Materials Technology Center (MTEC), National Science and Technology Development Agency, 114 Thailand Science Park, Phahonyothin Road, Klong Luang, Pathumthani 12120, Thailand; ^3^Faculty of Dentistry, Thammasat University, Pathumthani 12120, Thailand

## Abstract

Tissue engineering scaffold is a biological substitute that aims to restore, to maintain, or to improve tissue functions. Currently available manufacturing technology, that is, additive manufacturing is essentially applied to fabricate the scaffold according to the predefined computer aided design (CAD) model. To develop scaffold CAD libraries, the polyhedrons could be used in the scaffold libraries development. In this present study, one hundred and nineteen polyhedron models were evaluated according to the established criteria. The proposed criteria included considerations on geometry, manufacturing feasibility, and mechanical strength of these polyhedrons. CAD and finite element (FE) method were employed as tools in evaluation. The result of evaluation revealed that the close-cellular scaffold included truncated octahedron, rhombicuboctahedron, and rhombitruncated cuboctahedron. In addition, the suitable polyhedrons for using as open-cellular scaffold libraries included hexahedron, truncated octahedron, truncated hexahedron, cuboctahedron, rhombicuboctahedron, and rhombitruncated cuboctahedron. However, not all pore size to beam thickness ratios (PO : BT) were good for making the open-cellular scaffold. The PO : BT ratio of each library, generating the enclosed pore inside the scaffold, was excluded to avoid the impossibility of material removal after the fabrication. The close-cellular libraries presented the constant porosity which is irrespective to the different pore sizes. The relationship between PO : BT ratio and porosity of open-cellular scaffold libraries was displayed in the form of Logistic Power function. The possibility of merging two different types of libraries to produce the composite structure was geometrically evaluated in terms of the intersection index and was mechanically evaluated by means of FE analysis to observe the stress level. The couples of polyhedrons presenting low intersection index and high stress level were excluded. Good couples for producing the reinforced scaffold were hexahedron-truncated hexahedron and cuboctahedron-rhombitruncated cuboctahedron.

## 1. Introduction

Transplantation is a use of biological materials to replace the end state degenerative tissue [1, 2]. In general, natural biological materials such as autograft, allograft, and xenograft are commonly used as biological substitutes [3–7]. However, limited availability of harvested site, risks of host tissue rejection, and disease transmission are disadvantages [2, 8]. An alternative potential way to address these issues is to develop the synthetic biological substitute using concept of tissue engineering which can effectively mimic functions of tissue characteristics.

Tissue engineering is a multidiscipline which applies the principles of engineering and sciences to develop the biological substitutes, known as “scaffold”, which restore, maintain, or improve tissue functions [9–13]. The principle of tissue engineering involves the extraction of cells from biopsy and proliferates them in the scaffold which serve as a guide for neotissue formation [14, 15]. Successful scaffold should meet the biological and mechanical requirements to encourage cell formation. Some of the preferred requirements include sufficient strength, biocompatibility, appropriate porosity, adequate surface finish guaranteeing, and sterilisability [16–18]. Among these requirements, especially, pore size and scaffold architecture, have to be controlled in order to provide the appropriate scaffold porosity which encourages cellular activities inside the scaffold as well as to allow nutrients, oxygen, and waste to transport conveniently into or out of the scaffold [19, 20].

Scaffold can be extracted from natural resources such as coral [21] and animal bone [8] or can be synthesized using polymer processing techniques such as freeze drying, solvent casting, salt leaching, and so forth [10]. However, the drawbacks of scaffolds fabricated by these processes were the lack of uniform pore distribution and strength [22–25]. To overcome the drawbacks, the additive manufacturing (AM) techniques are used in the fabrication to control the scaffold properties, that is, pore size and beam thickness. The AM techniques use three-dimensional digital geometry to build the scaffold. As a result, the scaffold properties can then be predefined using the human-computer design interface, which is the computer aided design (CAD) [26]. Based on this technique, the properties of scaffold can be easily defined and controlled, if the primitive geometry is applied. Moreover, the technique allows to fabricate the customized scaffold which is not possible by other techniques [27, 28].

One way to facilitate the scaffold design process with the required porosity is to investigate the potential geometry which can be utilized as a three-dimensional scaffold. In advancement of the digital imaging, the design, and the manufacturing technologies, there have been many researches developing the analytical methods as well as proposing the geometries which can be used as scaffold libraries based on those technologies. For example, Hollister et al. [29] employed an image-based homogenization optimization to design the scaffold. Chua et al. [22, 30] presented the selection criteria for geometries which can be applied in tissue engineering libraries as well as performed manufacturing feasibility study for the proposed libraries based on additive manufacturing (AM) technologies. Naing et al. [27] developed the automatic algorithm to assemble polyhedron unit cell for modeling the customized scaffold that its shape is according to the anatomy obtained from medical imaging data. Fang et al. and Wettergreen et al. [31, 32] presented CAD models of scaffold libraries used in computer aided tissue engineering (CATE). Wettergreen et al. [33] also proposed creating scaffold used in vertebral body replacement and the method in combination of different scaffold libraries. More recently, Bucklen et al. [34] developed a library of tissue primitives and interfaces to be implemented in CATE.

Although, the literatures developed and proposed many scaffold libraries for tissue engineering, the other aspects of scaffold library still need to be investigated. These aspects included criteria in geometry selection for the open-cellular and the close-cellular scaffold library, minimum ratio between pore size and beam thickness which can be used for design process, relationship between scaffold parameters of each library, and porosity. Therefore, the authors aimed to conduct those aspects to help the design process of tissue engineering scaffold. In addition, today computer aided design/manufacturing (CAD/CAM) and AM have received much attention in scaffold fabrication and these technologies meet most of the scaffold design requirements, as a result, all aspects in this present study were considered along with these technologies.

## 2. Generation of Scaffold Library

### 2.1. Geometric Models

**Figure 1:**
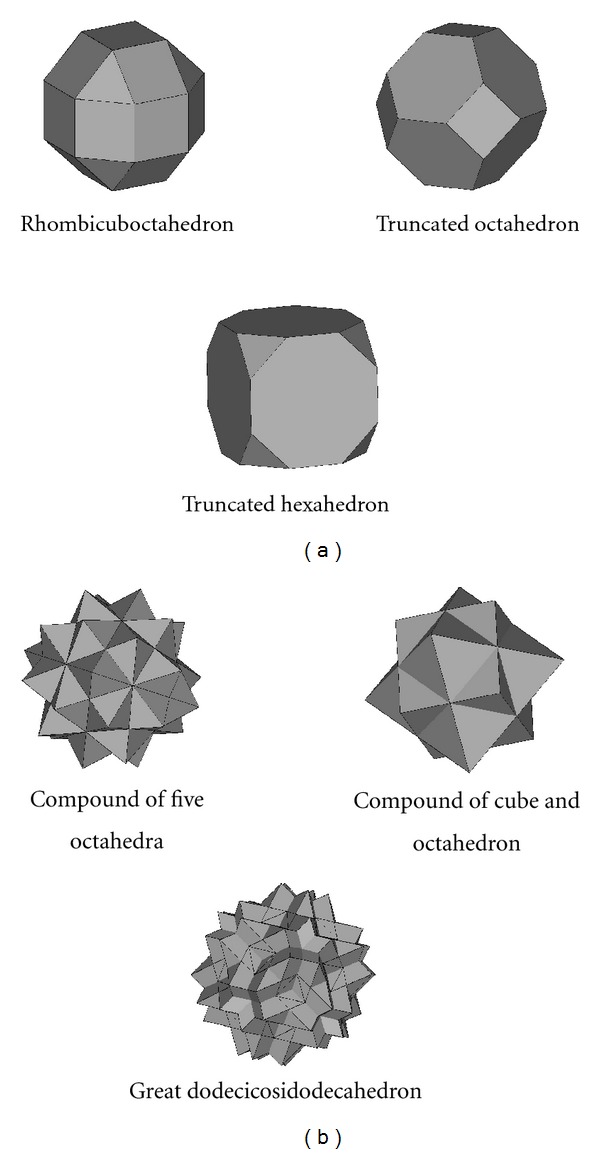
(a) Convex polyhedrons, (b) Nonconvex polyhedrons.

Stereolithography CAD models of 119 polyhedrons presented by Wenninger [35] were evaluated by their geometries for using as the scaffold libraries. These polyhedrons are generally categorized into (1) convex polyhedrons and (2) nonconvex polyhedrons. Some polyhedrons of each group are shown in Figure 1. Since the polyhedron may be enclosed by only a few (simple shape) or a great number of facet (complex shape), some polyhedrons may not be physically proper for tissue engineering applications for some reasons. Therefore, criteria for evaluating the polyhedrons were established.

In this study, three criteria were developed. The criteria A to C-1 were established for the close-cellular scaffold library whereas criteria C-2 was replaced with criteria C-1 to evaluate the open-cellular scaffold library.

### 2.2. Criteria

#### 2.2.1. Criteria A: Feasibility of Production Based on AM Technique

Laser-based AM technologies, that is, selective laser melting (SLM) [36], selective laser sintering (SLS) [37], and stereolithography (SL) [38], have been diversely employed for fabricating tissue engineering porous materials, especially metallic scaffolds [39]. Main limitations of the technologies include, for example, laser spot size and particle size of material [40]. As a result, the geometric details (edges and faces of polyhedron) of three-dimensional model should be larger than the described limitation in order to make possible for turning the details to physical prototype. Pore size for tissue scaffold ranges normally from 5–2250 micron depending on types of cell and tissue [41–43], for this reason, complex polyhedrons containing excessive geometric details, such as stellations and nonconvex forms, should not be used.

#### 2.2.2. Criteria B: Polyhedron Combinability

Single polyhedron is assembled into the other polyhedrons generating the matrix of scaffold. CATE is generally used to assist the assembly of three-dimensional CAD model by means of duplicating the polyhedron in Cartesian coordinate system (*x*-axis, *y*-axis, and *z*-axis). Therefore, the geometry of polyhedron should be symmetrical to provide sufficient contact of the interface between polyhedrons. Focusing on mechanical behavior, if the contact surface on one side of polyhedron is not conformed to the side of adjacent polyhedron, high stress level occurs at a junction between unit cells. This can easily lead to the failure of tissue engineering scaffold.

**Figure 2:**
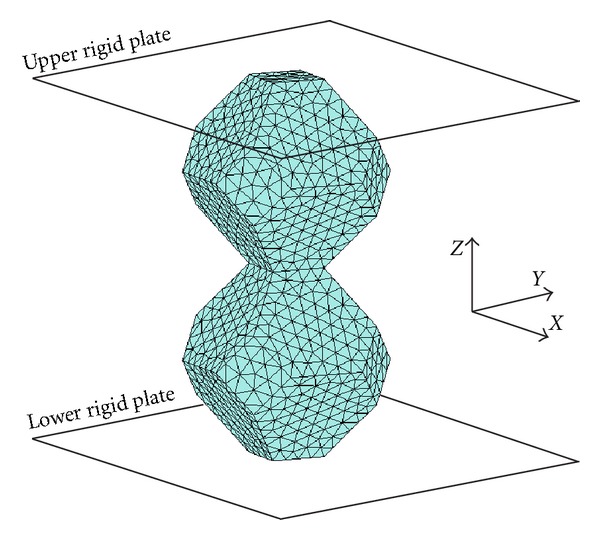
FE models for compressive strength analysis.

In order to evaluate the polyhedron combinability, the finite element (FE) models were created from the assembly of three-dimensional STL geometric models of polyhedron. FE meshes were generated in commercial FE preprocessor (Patran, MSC Software, Inc., USA). In this present study, only ten-node tetrahedron elements were used. Element size 1.0 mm was applied to all FE models which subsequently produced the numbers of elements employed in the analysis range from 2,670 to 16,570. Material properties for FE models were assumed to be homogenous, isotropic, and linearly elastic. The elastic properties of Titanium [44] were attributed (Elastic modulus = 110,000 MPa and Possion's ratio = 0.33) to FE models. For loading condition, axial load was applied to the polyhedrons to simulate the compression as shown in Figure 2. All FE models were preformed in commercial FE solver (Marc Mentat, MSC Software, Inc., USA).

The obtained equivalent von Mises (EQV) stress distributions on each of the considered polyhedrons in this study were compared. The polyhedrons having EQV high stress under described loading conditions were then eliminated.

#### 2.2.3. Criteria C: No Enclosed Pore after Assembly

**(i) Criteria C-1: Close-Cellular Tissue Engineering Scaffold**

**Figure 3:**
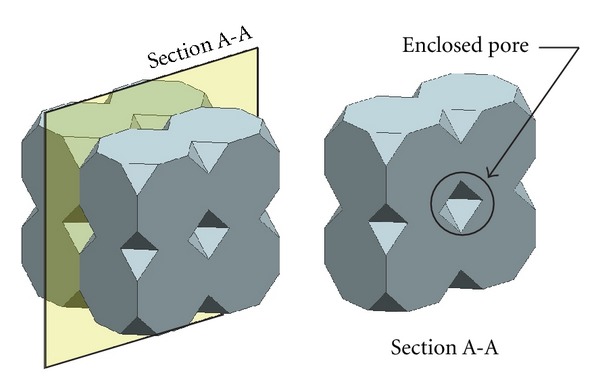
Enclosed pore inside close-cellular scaffold libraries.

The cells need certain pore size of tissue substitute to allow proliferation, and it is necessary for tissue engineering scaffolds to be a porous structure. Geometrically, the problem due to the assembly of some polyhedrons may generate the enclosed pore. The material removal in enclosed pores after the fabrication using AM techniques is then not possible. Consequently, polyhedrons have null porosity. To be able to eliminate the problem from polyhedrons, the three-dimensional CAD models of polyhedrons that passed Criteria B were assembled and evaluated for possible enclosed pore as shown in Figure 3.

**(ii) Criteria C-2: Open-Cellular Tissue Engineering Scaffold**

**Figure 4:**
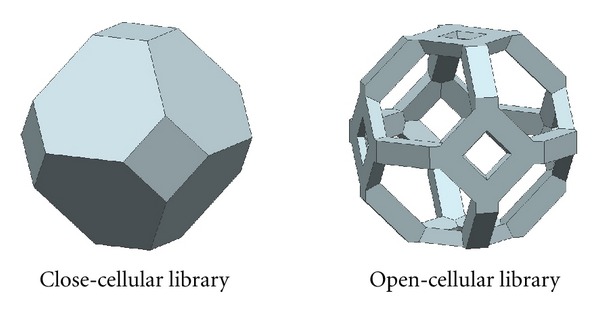
Close-cellular and open-cellular scaffold libraries of truncated octahedron.

**Figure 5:**
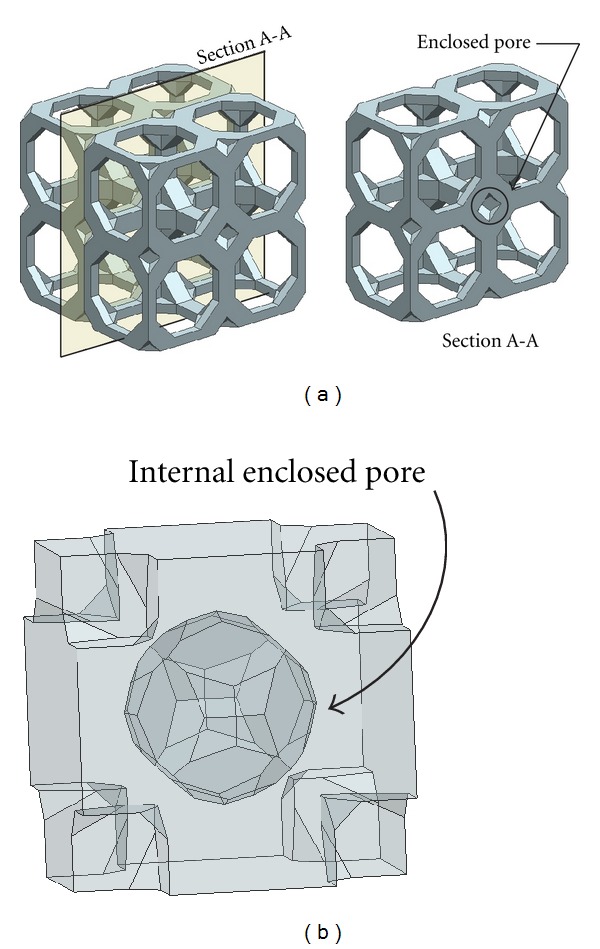
(a) Enclosed pore by surrounding cells, (b) enclosed pore inside the cell.

Apart from the close-cellular tissue engineering scaffolds, the wireframe of the polyhedrons can be thickened to make the open-cellular tissue engineering scaffolds as demonstrated in Figure 4. For open-cellular form of polyhedrons, the pores can be geometrically generated by two ways:

1. pore between polyhedrons (conventionally seen from assembly of close form polyhedrons), as shown in Figure 5(a) and
2. pore inside polyhedrons excessive dimension of beam thickness may also generate enclosed pore, as shown in Figure 5(b).

For better understanding of the effect of beam thickness dimension on the generation of enclosed pore, it is desirable to investigate how the ratio between pore size and beam thickness (PO : BT) influences the geometry of open-cellular polyhedrons. The evaluation was performed by creating three-dimensional CAD models of polyhedrons, which passed Criteria B based on various PO : BT values were 1 : 1, 2 : 1, 3 : 1, 4 : 1, 5 : 1, 6 : 1, 8 : 1, 10 : 1, and 12 : 1. The PO : BT values of each open-cellular form of polyhedrons which produced enclosed pore, would be excluded.

### 2.3. Evaluation Results

Criteria A eliminated stellations and nonconvex forms of polyhedrons. The remained polyhedrons were tetrahedron (P-1), octahedron (P-2), hexahedron (P-3), icosahedron (P-4), dodecahedron (P-5), truncated tetrahedron (P-6), truncated octahedron (P-7), truncated hexahedron (P-8), truncated icosahedrons (P-9), truncated dodecahedron (P-10), cuboctahedron (P-11), icosidodecahedron (P-12), rhombicuboctahedron (P-13), rhombicosidodecahedron (P-14), rhombitruncated cuboctahedron (P-15), rhombitruncated icosidodecahedron (P-16), snub cube (P-17), and snub dodecahedron (P-18).

In Criteria B, the stress analyses based on described loading conditions were employed for 18 polyhedrons. As shown in Table 1, the results revealed that the polyhedrons could be classified into three groups according to the stress levels. The first group contained the polyhedrons having very high EQV stress under the load which were tetrahedron (P-1), dodecahedron (P-5), truncated tetrahedron (P-6), and truncated icosahedrons (P-9). The second group contained the polyhedrons having moderate EQV stress which were octahedrons (P-2), icosahedron (P-4), truncated dodecahedron (P-10), icosidodecahedron (P-12), rhombicosidodecahedron (P-14), rhombitruncated icosidodecahedron (P-16), and snub dodecahedron (P-18). The last group contained the polyhedrons having relatively low stress compared to the others, which were hexahedron (P-3), truncated octahedron (P-7), truncated hexahedron (P-8), cuboctahedron (P-11), rhombicuboctahedron (P-13), rhombitruncated cuboctahedron (P-15), and snub cube (P-17). As the polyhedrons in the last group present the low stress level compared to the others, therefore, they were eligible passing Criteria B.

From the stress analysis, it can also be seen that the excluded polyhedrons were asymmetric as well as lack of interface for connecting itself to another one. Most of the polyhedrons with the low stress group were isotropic symmetry, except the snub cube (P-17). The isotropic symmetric polyhedrons are normally proffered by CAD software. This is because, in the polyhedron combination process, the isotropic symmetry CAD models require low memory and short computation time. As a result, even though snub cube (P-17) presented the low EQV stress, an open-cellular library of snub cube may lack of interface for combination. Snub cube (P-17) had better to be eliminated.

#### 2.3.1. Potential Close-Cellular Scaffold Library

**Figure 6:**
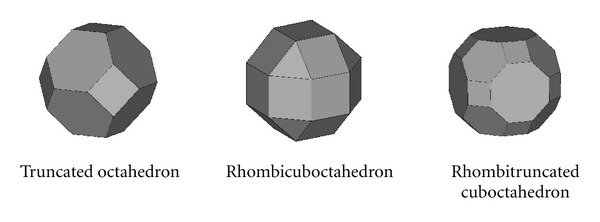
Potential close-cellular libraries.

Criteria C-1 was employed to evaluate the polyhedrons which could be the close-cellular libraries.  Figure 6 shows the potential close-cellular scaffold libraries included truncated octahedron (P-7), rhombicuboctahedron (P-13), and rhombitruncated cuboctahedron (P-15).

#### 2.3.2. Potential Open-Cellular Scaffold Library

**Figure 7:**
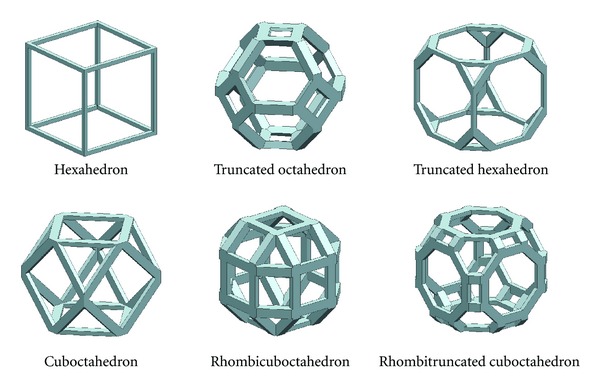
Potential open-cellular libraries.

Criteria C-2 was employed to evaluate the polyhedrons which could be the open-cellular libraries. The potential open-cellular scaffold libraries are shown in Figure 7.  Table 2 shows the analytical results of the influence of PO : BT ratio to the geometry of the libraries as well as the generation of enclosed pore. From the table, the completeness of geometry could be classified into three groups as follows.

- Group A: the geometry was perfect, containing all components of polyhedrons.
- Group B: the geometry lost some components of polyhedrons, no enclosed void found after assembly.
- Group C: the geometry contained the enclosed void after assembly.

## 3. Porosity of Scaffold

The porosity is determined by the relationship between the volume of scaffold material and the apparent scaffold volume (bounding volume of scaffold). The mathematical formula relating to the porosity calculation is given in the following equation:

(1) $$\begin{array}{c} \phi =1-\frac{{\text{Volume}}_{\text{scaffold material}}}{{\text{Volume}}_{\text{apparent scaffold}}}. \end{array}$$

The porosity ranges from “0” to “1”. The nearly “0” value means the scaffold is dense (solid) whereas the nearly “1” value means the scaffold is more porous.

### 3.1. Close-Cellular Scaffold Library

**Figure 8:**
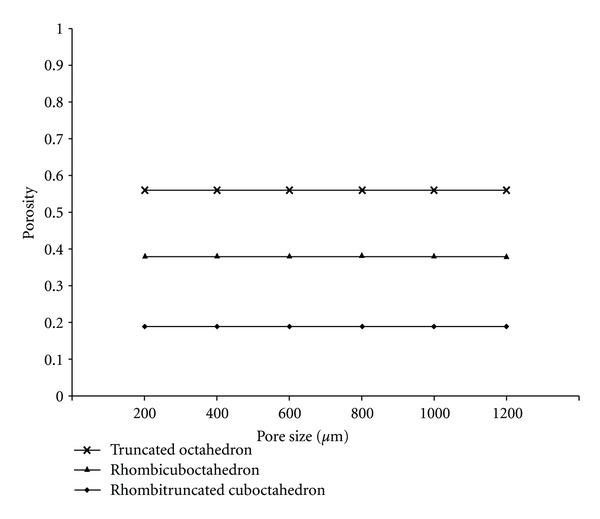
Relationship between pore size and porosity of close-cellular scaffold libraries.

As shown in Figure 8, porosity of the close-cellular scaffold libraries was constant regardless of pore size. Truncated octahedron had the highest degree of porosity whereas rhombitruncated cuboctahedron had the lowest degree of porosity.

### 3.2. Open-Cellular Scaffold Library

**Figure 9:**
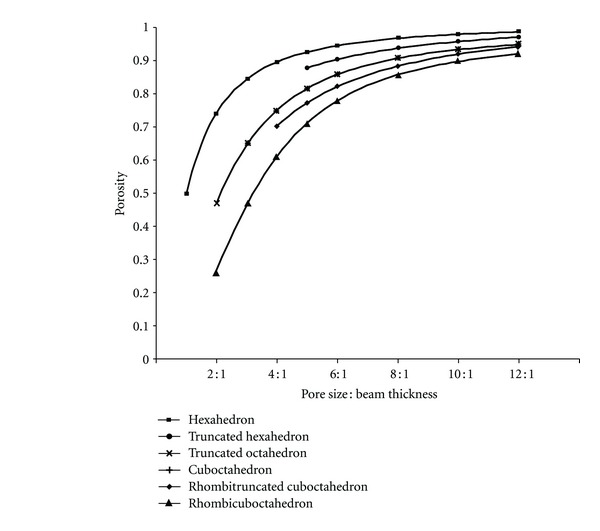
Relationship between PO : BT and porosity of open-cellular scaffold libraries.

For the open-cellular scaffold libraries, the PO : BT ratio in Group A and B was analyzed for the porosity. As shown in Figure 9, the order of porosities ranged from high to low degree in all PO : BT ratios was: hexahedron (P-3), truncated hexahedron (P-8), truncated octahedron (P-7), cuboctahedron (P-11), rhombitruncated cuboctahedron (P-15), and rhombicuboctahedron (P-13). From the chart, it can be seen that truncated octahedron (P-7) and cuboctahedron (P-11) had the equivalent values of porosity.

The relationship between PO : BT ratio and porosity could be determined mathematically using the regression analysis. Various regression functions were trialed to observe the correlation between both parameters. Many functions could well describe the relationship since the coefficient of correlations (*r*) of these functions was high. Nevertheless, some regression functions are rather complex and contain many constants, it is therefore difficult to be used. In this present study, Logistic Power function was used to describe the relationship which can be written in the following form:

(2) $$\begin{array}{c} y=\frac{a}{1+{\left(x/b\right)}^{c}}. \end{array}$$

In (2), *a*, *b*, and *c* are constants whereas *x* and *y* are independent and dependent variables, respectively.  Table 3 shows the set of equations describing the relationship between PO : BT ratios and porosity of each open-cellular library.

## 4. Libraries Merging Analysis

Mechanically, the high porosity scaffold has the lower strength than its null porosity (solid) structure. This is because the solid material is removed, thus the subject volume to the loads becomes less. The high porosity scaffold is nevertheless proper for regeneration environment, since there is a large space for fluid containing necessary substances required for cell growth to circulate into and away from the scaffold. The designed scaffold having high porosity may not cope with the strength under physiological loads. Additionally, some organs have more than single mechanical properties, the scaffold may require differently the strength in each location. The different scaffold libraries may be selected and composing up the entire scaffold structure [33]. In order to investigate the feasibility on merging two different polyhedrons, the geometric mismatch of polyhedron interfaces and the stress exhibiting on the interface under axial loads were carried out.

### 4.1. Geometric Mismatch Analysis

The analysis of geometric mismatch of polyhedron interfaces was performed by placing two of the three-dimensional CAD models of scaffold libraries contiguously. Low geometric mismatch refers to the large common interface area whereas high geometric mismatch refers to the small common interface area. The common interfaces can be measured as intersection index in percentage using the following equations

(3) Intersection  index=(Contact  Area  Library  A)∩(Contact  Area  Library  B)Possible  Maximum  Interface  Area ×100.

A hundred percent common intersection index indicates the perfect match of interface of both scaffold libraries. Good example of perfect matching interface is the merging of the isometric symmetrical polyhedrons such as truncated octahedron (P-7). In addition, zero percent intersection index implies no intersection of both interfaces.

According to the analysis result, the merging of two different close-cellular scaffold libraries was geometrically possible because of the high interface area. The interface area of the close-cellular scaffold library is always higher than that of the open-cellular scaffold library. Focusing on the analytic results of open-cellular scaffold libraries, Table 4 shows the results of geometric mismatch analysis. The results can be classified into three groups as follows.

**(i) Group I: Not Possible Couple**

The couples in this group could not be joined together due to lack of common faces. The couples in Group I are listed as follows:

1. hexahedron-truncated octahedron,
2. hexahedron-rhombicuboctahedron,
3. hexahedron-rhombitruncated cuboctahedron,
4. cuboctahedron-truncated octahedron,
5. truncated hexahedron-truncated octahedron,
6. truncated hexahedron-cuboctahedron,
7. truncated hexahedron-rhombicuboctahedron,
8. truncated hexahedron-rhombitruncated cuboctahedron.

**Figure 10:**
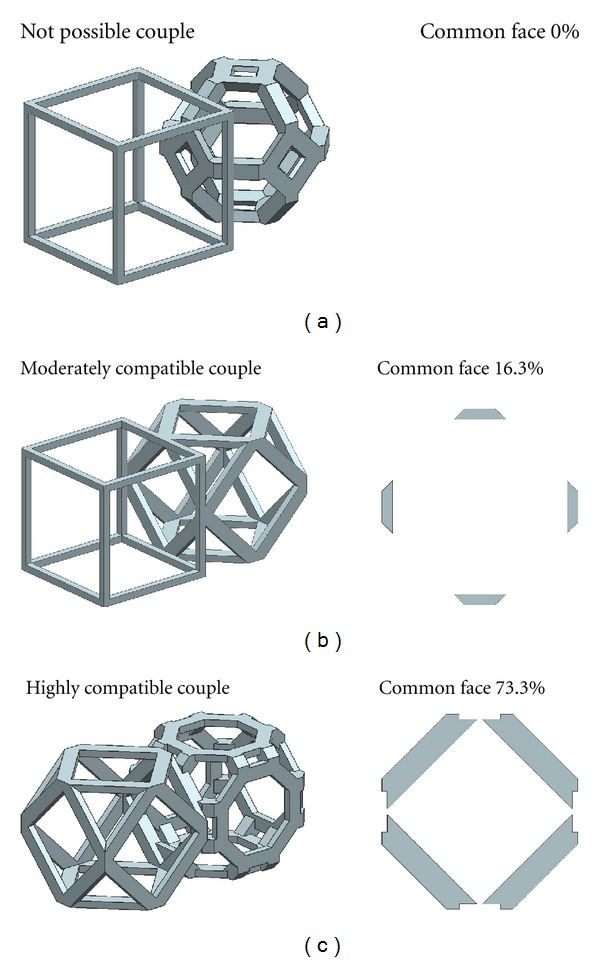
Characteristics of common face of each couple group.

Figure 10(a) shows one of the couples in Group I.

**(ii) Group II: Moderately Compatible Couple**

The couples in this group had partly contact area. The couples in Group II included

1. hexahedron-cuboctahedron,
2. cuboctahedron-truncated hexahedron,
3. cuboctahedron-rhombicuboctahedron,
4. truncated octahedron-rhombicuboctahedron,
5. rhombicuboctahedron-rhombitruncated cuboctahedron.

Figure 10(b) shows one of the couples in Group II.

**(iii) Group III: Highly Compatible Couple**

The couples in this group could be properly joined together. Most of contact surfaces of one library almost completely contact to another library, therefore the high intersection index could be observed. The couples in this group were

1. hexahedron-truncated hexahedron,
2. cuboctahedron-rhombitruncated cuboctahedron.

Figure 10(c) shows one of the couples in Group III.

### 4.2. Stress Analysis

**Figure 11:**
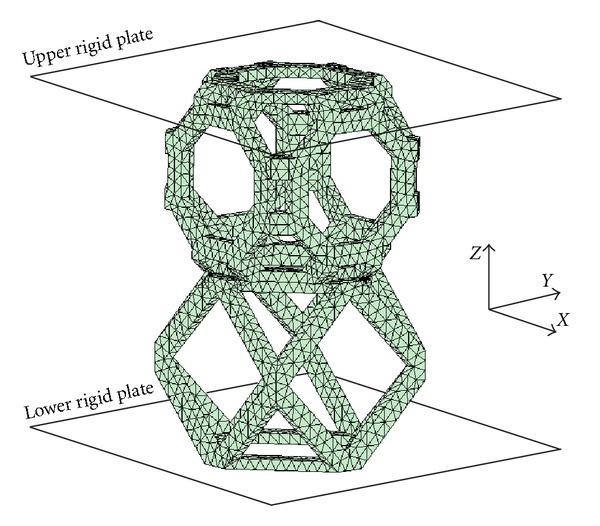
FE model for library merging analysis.

CAD models of the couples in Group II and Group III were used to generate ten-node tetrahedral elements for FE analysis. Rigid plates were created at both ends of the FE models. In each FE model, the rigid plates were compressed by 0.02 percent strain to examine the stress level at junction between two libraries.  Figure 11 shows one of the FE models.

**Figure 12:**
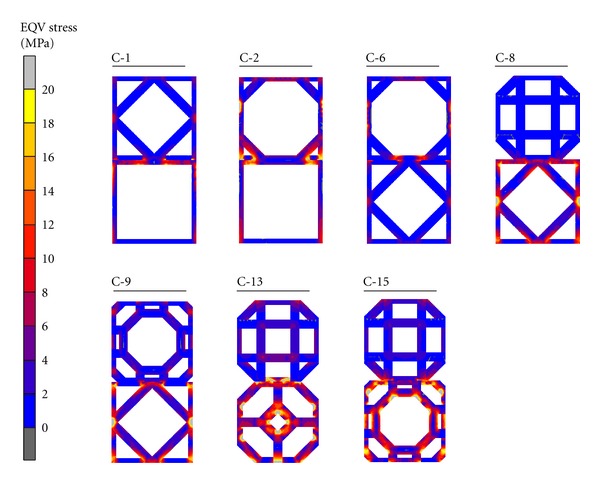
EQV stress exhibited on each couple.

Table 4 and Figure 12 show the results of stress analysis. The couples in Group II revealed higher stress than most of couples in Group III. Truncated octahedron-rhombicuboctahedron presented the highest stress whereas cuboctahedron-truncated hexahedron presented the lowest EQV stress among the couples. Additionally, level of the EQV stress was irrelevant to the intersection index.

## 5. Discussion

Three-dimensional tissue engineering scaffold is considered to be a key element in success of tissue regeneration. The tissue engineering scaffold can be extracted from the natural substances or synthesized by various polymer processing techniques [10, 21]. By means of these techniques, lack of uniformity and strength of scaffold are problems [22–25]. A good alternative fabrication technique is applying the digital system, that is, CAD and AM, to avoid those problems. Based on the digital system, the scaffold parameters can be controlled in the fabrication process. One way to develop the scaffold libraries, the primitive geometries such as polyhedrons are suitable to be utilized.

Although, there are various available polyhedrons, not all of them are suited for using in tissue engineering applications. Therefore, the criteria for selecting the proper polyhedrons to be used as the scaffold libraries for tissue engineering applications were developed in this present study. Three criteria were proposed for the open-cellular and the close-cellular scaffolds in terms of the limitation of fabrication devices and geometry. Each polyhedron required the orderly assessment to all criteria. For the first criteria (Criteria A), feasibility of production based on AM, the complex polyhedrons were eliminated as some components may be suppressed during the fabrication due to the limitations of AM devices in fabrication small dimensional scaffold. For example, fabrication of bone scaffold may require pore size ranges from 100–700 micron [36].

Criteria B and Criteria C were established to evaluate the geometrical limitations of the polyhedrons after assembly (making up the scaffold). The evaluation was based on FE method which is widely accepted as a useful technique to evaluate or predict the biomechanical behavior of biological substitutes [19], implants [44], and organs [45] under certain loading conditions. According to the results, it can be obviously noticed that the polyhedrons could be categorized into three groups relevant to the exhibited stress level. For the polyhedrons in the high EQV and moderate EQV stress groups, they assembled together for making up the scaffold by connecting each other by vertex or edge. This relates to the effect of interface between the polyhedron. In engineering terms, the less interfaces produce the higher EQV stress concentration around the junction between unit cells under axial loading condition. Therefore, the assembly by connecting vertex-to-vertex or edge-to-edge can be at risk. On the other hand, the polyhedrons connect to each other by face so that they have the large interface. The large interface normally allows the force to distribute throughout the interface, the EQV stress level subsequently reduces.

According to the evaluation results, the polyhedrons which are proper for using as the open-cellular and the close-cellular scaffold libraries were isotropic symmetry: hexahedron (P-3), truncated octahedron (P-7), truncated hexahedron (P-8), cuboctahedron (P-11), rhombicuboctahedron (P-13), and rhombitruncated cuboctahedron (P-15). The results were also compared with the ones in previous study [22, 30]; it was found that the proposed polyhedrons for utilizing as scaffold libraries are almost similar. However, the mechanical aspect has not been assessed in the previous study. In the previous study, the proposed polyhedrons also included prisms which are triangular prism, hexagonal prism, and octagonal prism. However, these prisms are asymmetric and may be complicated to join together using CATE automatic software system generating the scaffold. Concept of the CATE is that the scaffold libraries are duplicated along Cartesian axis inside the specified boundary (volume). Some CATE software requires the symmetry scaffold libraries to reduce the time required for software computation. In addition, the previous study suggested that Archimedean dual called rhombic dodecahedron is suitable for scaffold library [22, 30]. In the present investigation, the Archimedean duals are beyond the scope, and the evaluation results of this present study were slightly different from that previous investigation.

**Figure 13:**
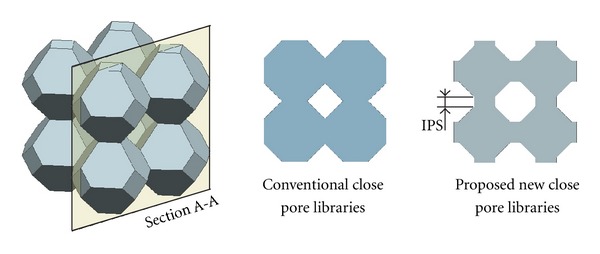
Conventional and proposed new close pore scaffold library.

The porosity of the scaffold depends on the amount of apparent volume space occupied by material. The close-cellular scaffold libraries presented the constant porosity no matter of the increasing or the decreasing of pore size. The pore size of close-cellular scaffold libraries could be determined by the void among adjacent polyhedrons. Thus, the size of polyhedron influenced directly on the volume of void space. Even though the large polyhedron produced the large pore size, unfortunately, the increase of pore size required the increase of polyhedron size by the same amount of volume. From the analysis, some polyhedrons were not included to be used as close-scaffold libraries because their assembly generated the enclosed pore. After fabrication process by AM technique, it is subsequently impossible for material to be removed. The excessive material is trapped inside the enclose pores. The scaffold finally produces almost null porosity. In order to effectively raise the porosity of the close-cellular scaffold, the interconnected pore structure (IPS) can be used to join between polyhedrons as shown in Figure 13. By attaching the polyhedrons using IPS, the porosity increases according to the increase of IPS length.

Some libraries of the open-cellular scaffold with some PO : BT ratios could not be fabricated, because they contained the enclosed voids inside the scaffold. Exactly similar reason to the enclosed pore found in the close-cellular scaffold is that the excessive material is trapped and cannot be removed. For the availability score of the open-cellular scaffold libraries, the score is given as “2”, “1”, or “0” if the PO : BT ratios of each scaffold were in Group A, Group B, and Group C, respectively. The libraries having higher score provided the better availability for fabrication in a wide range of PO : BT ratio. Hexahedron (P-3) was considered to be the best library as its score was higher than those of the others. The other libraries which had the lower scores were truncated octahedron (P-7) and cuboctahedron (P-11), rhombicuboctahedron (P-13), rhombitruncated cuboctahedron (P-15), and truncated hexahedron (P-8), respectively. This availability sequence can be geometrically explained that the polyhedrons having few edges and faces require only few beams to compose the scaffold. Therefore, in case of the large beam generated, there is less possibility that the beam overlaps to other beams. Diversely, the polyhedrons having many edges and faces, such as rhombitruncated cuboctahedron (P-15), contain many beams. The beams may overlap in which can generate enclosed pore.

**Figure 14:**
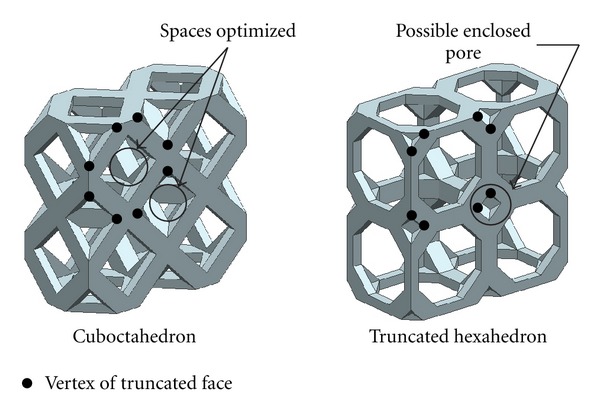
Optimized space and possible enclosed pore resulting from position of vertex of truncated face of polyhedrons.

The polyhedron without truncated faces allows the space inside the scaffold to be maximized leaving no space between the polyhedrons. For this reason, the small pore between polyhedrons is absent. However, if the space inside the scaffold is not maximized, pore between polyhedrons is existed. The size of pore depends on the position and angle of the truncated faces. For example, the structure of truncated hexahedron (P-3) is almost similar to cuboctahedron (P-11), but the position of truncated faces is different. The vertices of truncated faces in the cuboctahedron (P-11) are mid-edge of surface whereas the vertices of truncated faces in the truncated hexahedron (P-8) are shifted toward the corner-edge of surface, as shown in Figure 14. The vertices of truncated face located at the mid-edge of surface are optimized, that is, cuboctahedron (P-11), the space between the polyhedrons and inside peripheral of polyhedrons. Truncated hexahedron (P-8) presents the space between polyhedrons significantly smaller than the space inside polyhedrons. The large beam for open-cellular truncated hexahedron (P-8) library may generate the enclosed pore between polyhedrons. For this reason, the availability score of cuboctahedron (P-11) was higher than the availability score of truncated hexahedron (P-8).

The Logistic Power function describing the relationship is considered to be effective and can be used to assist the scaffold design since the coefficient of correlation was nearly “1.0”. From the Figure 9, it can be noticed that the porosity of the open-cellular scaffold library was strongly influenced by the PO : BT ratio.

Different libraries can be assembled together, making the reinforced structure, to meet the mechanical and biological requirements of host tissue. Despite the fact that the combination of different polyhedrons is a good choice, the compatibility in terms of mechanics and geometry needed to be under consideration. From the geometric mismatch analysis, the higher intersection index was found in the combination between library and different library geometry, but geometrically subset of another one, such as hexahedron-truncated hexahedron, and rhombicuboctahedron-rhombitruncated cuboctahedron. Although, the geometric details are slightly different, the main components remain similar. Therefore, the common interface of polyhedrons in those couples was possible to connect to each other.

Most of the combinations in the present study yielded no common interface between polyhedrons or have only partial common interface, as the intersection index was null or low. Nevertheless, to combine the couples having without or partial common interface, the torus portion proposed by Wettergreen et al. [33] may be used. The cell merging analysis using FE method showed that the couples in Group III presented the lower EQV stress than the other couples. This can be explained that the intersection index agree with the EQV stress level. However, the EQV stress depends not only on the intersection index, but also on the stiffness of the structure. In general, the porosity corresponds inversely to the stiffness [46]. The scaffold libraries containing vertical beam are led to higher stiffness of the structure. Since rhombicuboctahedron (P-13) was in the low porosity group, it tends to stiffer than the libraries in the other groups. From the results, because of the high stiffness of rhombicuboctahedron (P-13), the EQV stress at the junction of the couple containing rhombicuboctahedron (P-13) was high. The possible couples for utilizing as the reinforced scaffold included hexahedron-truncated hexahedron, cuboctahedron-truncated hexahedron, and cuboctahedron-rhombitruncated cuboctahedron.

**Figure 15:**
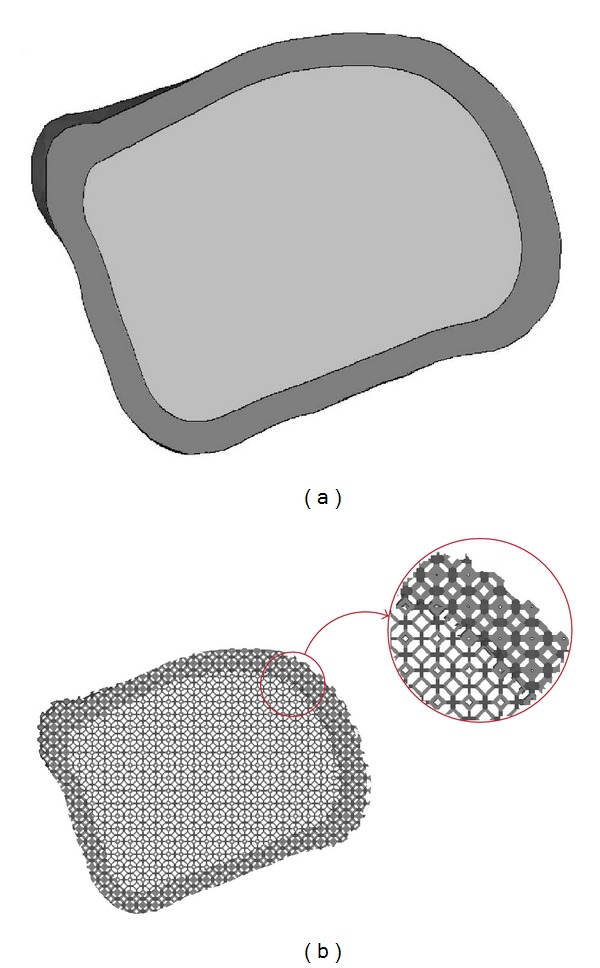
(a) CAD model of cortical and tubercular layer of bone, (b) variation of PO : BT scaffold generated based on the CAD model.

Furthermore, since the anatomical geometry of some organs, for example long bone has gradient distribution of pore size, the concept of heterogeneous scaffold (functionally graded scaffold, FGS) has therefore been recently a new trend in tissue engineering [46]. The scaffold stiffness can be controlled by the size of pore. The lower pore size is led to higher stiffness of the structure. From the results in the present study, in order to simplify the design of FGS for long bone defect, the units block inside the scaffold may be varied. The lower PO : BT scaffold may be applied to the layer of cortical bone whereas the higher PO : BT scaffold may be applied to the tubercular layer.  Figure 15 shows an example of the bone scaffold composing up by integrating different PO : BT ratios to make it suitable for cortical and tubercular layers.

## 6. Conclusions

This study presented the evaluation of 119 polyhedrons for using as the open-cellular and the close-cellular scaffold libraries. Three proposed criteria were used to evaluate each polyhedron.

1. Criteria A was based on the manufacturing consideration. The complex polyhedrons containing excessive geometric details, which may not be possible to fabricate due to the limitations of AM device, were eliminated.
2. Criteria B was based on the geometric and mechanical strength consideration. The joining polyhedrons that result high EQV stress at the junction between polyhedrons under loading were excluded. Most of the excluded polyhedrons joined to each other by only a small area interface.
3. Criteria C was based on the geometric consideration. The polyhedrons having the enclosed pore after assembly of polyhedrons rejected. This is to avoid the impossibility of material removal.

According to the analysis, the proper polyhedrons for using as the close-cellular library included truncated octahedron (P-7), rhombicuboctahedron (P-13), rhombitruncated cuboctahedron (P-15), and snub cube (P-17). For open-cellular libraries, the proper polyhedrons included hexahedron (P-3), truncated octahedron (P-7), truncated hexahedron (P-8), cuboctahedron (P-11), rhombicuboctahedron (P-13), and rhombitruncated cuboctahedron (P-15). In addition, some of PO : BT ratios of open-cellular scaffold libraries were not proper as they had large beam thickness and small pore size which could generate the enclosed pore. The relationships between pore size and close-cellular library were constant no matter at what large the pore size was. The relationship between PO : BT ratio and porosity of the open-cellular scaffold libraries was displayed in the Logistic Power function. Merging two different types of libraries was also evaluated geometrically and mechanically in terms of intersection index and EQV stress level. Good couples for merging were hexahedron-truncated hexahedron and cuboctahedron-rhombitruncated cuboctahedron.

Finally, by this way of investigation, the results from the present study would beneficially assist the tissue engineering scaffold design process.

## Figures and Tables

**Table 1:** EQV stress of each polyhedron assembly model.

Polyhedron	Axial stress (MPa)
Tetrahedron (P-1)	1266.5
Octahedron (P-2)	818.7
Hexahedron (P-3)	117.9
Icosahedron (P-4)	716.0
Dodecahedron (P-5)	1534.0
Truncated tetrahedron (P-6)	1040.8
Truncated octahedron (P-7)	368.8
Truncated hexahedron (P-8)	229.3
Truncated icosahedrons (P-9)	1042.3
Truncated dodecahedron (P-10)	648.3
Cuboctahedron (P-11)	253.1
Icosidodecahedron (P-12)	754.9
Rhombicuboctahedron (P-13)	178.4
Rhombicosidodecahedron (P-14)	680.5
Rhombitruncated cuboctahedron (P-15)	153.4
Rhombitruncated icosidodecahedron (P-16)	501.2
Snub cube (P-17)	332.5
Snub dodecahedron (P-18)	958.5

**Table 2:** Evaluation results.

Library	PO : BT								
	1 : 1	2 : 1	3 : 1	4 : 1	5 : 1	6 : 1	8 : 1	10 : 1	12 : 1
Hexahedron (P-3)	A	A	A	A	A	A	A	A	A
Truncated octahedron (P-7)	C	B	B	A	A	A	A	A	A
Truncated hexahedron (P-8)	C	C	C	C	A	A	A	A	A
Cuboctahedron (P-11)	C	C	A	A	A	A	A	A	A
Rhombicuboctahedron (P-13)	C	B	B	B	A	A	A	A	A
Rhombitruncated cuboctahedron (P-15)	C	C	C	B	A	A	A	A	A

A: Group A, B: Group B, C: Group C.

**Table 3:** A set of equations described the relationship between PO : BT ratio and porosity.

Library	Equation (*x*: PO : BT ratio, *y*: porosity)	Correlation (*r*)
Hexahedron	*y* = 1.01/(1 + (*x*/1.02)^−1.50^)	0.999
Truncated octahedron	*y* = 0.99/(1 + (*x*/2.12)^−1.79^)	0.999
Truncated hexahedron	*y* = 1.03/(1 + (*x*/1.14)^−1.18^)	0.997
Cuboctahedron	*y* = 0.99/(1 + (*x*/2.12)^−1.79^)	0.999
Rhombicuboctahedron	*y* = 0.97/(1 + (*x*/3.13)^−2.15^)	0.999
Rhombitruncated cuboctahedron	*y* = 1.02/(1 + (*x*/2.41)^−1.54^)	0.999

**Table 4:** Interface Index and EQV stress level of each open-cellular couple.

Couple	Intersection index (percent)	EQV stress (MPa)
Hexahedron-cuboctahedron (C-1)	16.3	33.8
Hexahedron-truncated hexahedron (C-2)	54.9	30.3
Hexahedron-truncated octahedron (C-3)	0.0	—
Hexahedron-rhombicuboctahedron (C-4)	0.0	—
Hexahedron-rhombitruncated cuboctahedron (C-5)	0.0	—
Cuboctahedron-truncated hexahedron (C-6)	16.3	23.6
Cuboctahedron-truncated octahedron (C-7)	0.0	—
Cuboctahedron-rhombicuboctahedron (C-8)	14.5	24.4
Cuboctahedron-rhombitruncated cuboctahedron (C-9)	73.3	29.5
Truncated hexahedron-truncated octahedron (C-10)	0.0	—
Truncated hexahedron-rhombicuboctahedron (C-11)	0.0	—
Truncated hexahedron-rhombitruncated cuboctahedron (C-12)	0.0	—
Truncated octahedron-rhombicuboctahedron (C-13)	37.7	56.1
Truncated octahedron-rhombitruncated cuboctahedron (C-14)	0.0	—
Rhombicuboctahedron-rhombitruncated cuboctahedron (C-15)	14.7	40.2
